# P-2045. Humoral Response induced by Three-Dose Vaccination Schemes Against SARS-CoV-2 in Pediatric Hematopoietic Stem Cell Transplant Recipients: Experience from a Middle-Income Country

**DOI:** 10.1093/ofid/ofae631.2201

**Published:** 2025-01-29

**Authors:** Andrés Soffia, M Nicole Le Corre, Juan Pablo Torres, M Cecilia Vizcaya, Constanza Martinez-Valdebenito, Natalia González, Paula Catalán, Paulina Donoso, Aldo Barrera

**Affiliations:** Pontificia Universidad Católica de Chile, Santiago, Region Metropolitana, Chile; Pontificia Universidad Católica de Chile, Santiago, Region Metropolitana, Chile; Department of Pediatrics, University of Chile, Santiago, Region Metropolitana, Chile; Pontificia Universidad Católica de Chile, Santiago, Region Metropolitana, Chile; Departamento de Enfermedades Infecciosas e Inmunología Pediátricas, Escuela de Medicina, Pontificia Universidad Católica de Chile., santiago, Region Metropolitana, Chile; Universidad de Chile, Santiago, Region Metropolitana, Chile; Pontificia Universidad Católica de Chile, Santiago, Region Metropolitana, Chile; Hospital Dr. Luis Calvo Mackenns, Santiago, Region Metropolitana, Chile; Pontificia Universidad Católica de Chile, Santiago, Region Metropolitana, Chile

## Abstract

**Background:**

Hematopoietic Stem Cell Transplant (HSCT) recipients are at high risk for severe COVID-19, requiring effective vaccination strategies. SARS-CoV-2 inactivated vaccines elicit lower levels of neutralizing antibodies (NAbs) compared to mRNA vaccines and have been little studied in immunocompromised children. However, low- and middle-income countries predominantly use inactivated vaccines. Aim: to explore the immunogenicity of different COVID-19 vaccination strategies in pediatric HSCT recipients from Chile, where both vaccine types are used.

**Figure:**
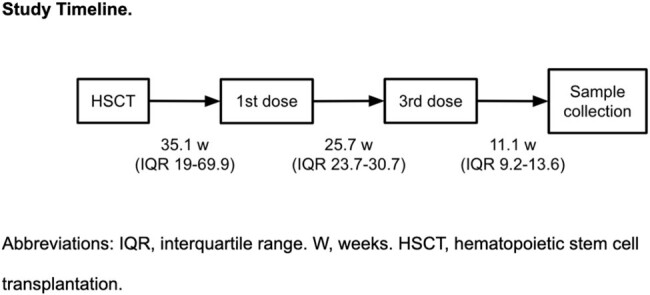

**Methods:**

Multicenter cross-sectional study of pediatric HSCT recipients, previously immunized with 3-dose regimens based on CoronaVac/Sinovac (CV) and BNT162b2/Pfizer-BioNTech (BNT) vaccines >=3 months post-transplant. NAbs against the original Wuhan strain and the Omicron XBB1.5 variant were evaluated 8-12 weeks after the 3rd dose using a pseudovirus-based assay. A positive Nab titer was defined as a neutralizing activity of at least 50% (ID50) at a 1:160 dilution. Safety/reactogenicity outcomes were recorded.

**Figure:**
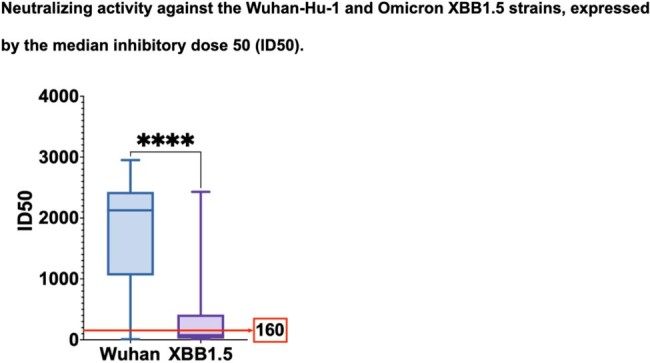

**Results:**

27 pediatric HSCT recipients were included (median age 11.9 y), of whom 21 were vaccinated with 2 doses of CV followed by a BNT booster, 1 with 2 doses of BNT followed by a CV booster and 5 with 3 BNT doses. At 11.1 weeks (IQR 9.2-13.6) after the 3rd dose, 84% reached NAb positivity for the Wuhan strain, while only 40% reached it for the XBB1.5 variant (P< 0.005). Neutralizing capacity was higher against Wuhan compared to XBB1.5, with a median ID50 of 2127 vs 75.9 (P< 0.0001). NAb-positive patients for the Wuhan strain had higher IgG levels (median 693 mg/dL vs 371.5 mg/dL in non-responders, P< 0.05). No significant differences were found in neutralizing capacity based on the scheme administered. In a multivariate linear regression, the CD19 count predicted neutralization capacity. No serious adverse events were reported.

**Figure:**
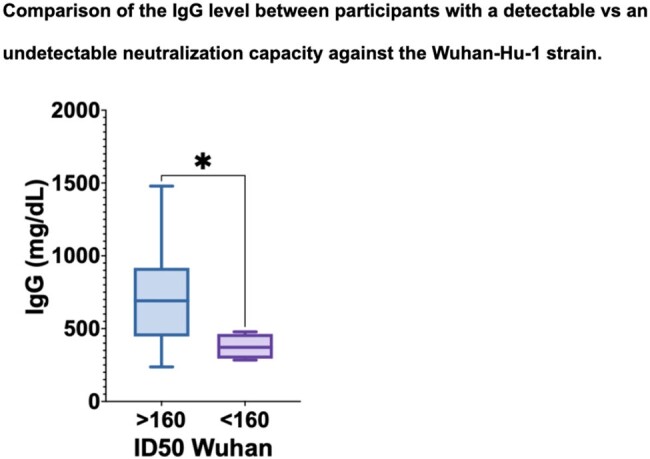

**Conclusion:**

No differences were observed in neutralizing capacity based on the vaccines administered, supporting the use of heterologous schemes including both inactivated and mRNA vaccines in HSCT pediatric recipients. Neutralizing capacity was higher against Wuhan than XBB1.5, emphasizing the importance of booster doses of bivalent vaccines that target this variant.

**Disclosures:**

All Authors: No reported disclosures

